# Sulforaphane Inhibits the Acquisition of Tobacco Smoke-Induced Lung Cancer Stem Cell-Like Properties *via* the IL-6/ΔNp63α/Notch Axis

**DOI:** 10.7150/thno.33812

**Published:** 2019-07-09

**Authors:** Chunfeng Xie, Jianyun Zhu, Ye Jiang, Jiaqi Chen, Xueqi Wang, Shanshan Geng, Jieshu Wu, Caiyun Zhong, Xiaoting Li, Zili Meng

**Affiliations:** 1 Department of Nutrition and Food Safety, School of Public Health, Nanjing Medical University, Nanjing, Jiangsu, 211166, China.; 2 Suzhou Digestive Diseases and Nutrition Research Center, North District of Suzhou Municipal Hospital. The Affiliated Suzhou Hospital of Nanjing Medical University, Suzhou, Jiangsu, 215008, China.; 3 Department of Food and School Hygiene, Taizhou Municipal Center for Disease Control and Prevention, Taizhou, Zhejiang, 318000, China.; 4 Center for Global Health, School of Public Health, Nanjing Medical University, Nanjing, Jiangsu, 211166, China.; 5 Department of Respiratory Medicine, Huai'an First People's Hospital, Nanjing Medical University, Huai'an, 223300, Jiangsu, China.

**Keywords:** tobacco smoke, lung cancer stem cells, ΔNp63α, Notch pathway, IL-6, sulforaphane

## Abstract

**Background:** Tobacco smoke (TS) critically contributes to the development of lung cancer; however, the underlying molecular mechanisms remain unclear. The induction of cancer stem cells (CSCs) by TS represents an early event in tumor initiation. The lung cancer-related gene ΔNp63α is highly expressed in epithelial tissues and drives tumor formation and cancer stem cell properties. This study investigated the role of ΔNp63α in the long-term acquisition of TS-induced lung CSC-like properties.

**Methods:** The expression levels of ΔNp63α, lung CSC markers, and interleukin (IL)-6 in lung carcinoma specimens were determined by western blotting and enzyme linked immunosorbent assays. Human bronchial epithelial (HBE) cells were chronically exposed to 2 % cigarette smoke extract for 55 passages, following which colony formation capacity, expression of proteins associated with malignant transformation, lung CSC markers, and tumor incidence were investigated. The effects of ΔNp63α on long-term TS exposure-induced lung CSC-like properties and Notch activation were analyzed using tumorsphere formation ability, immunofluorescence assays, luciferase reporter assays, and western blotting. The roles of IL-6 on chronic TS exposure-induced lung CSC-like properties and ΔNp63α expression were also examined. Moreover, the effects of sulforaphane (SFN) on TS-transformed lung CSC-like properties, IL-6 and ΔNp63α expression, and Notch signaling were investigated *in vitro* and *in vivo*.

**Results:** Higher levels of ΔNp63α were observed in the lung cancer tissues of smokers than in those of non-smokers, whereas ΔNp63α was positively correlated with CD133 and Oct4 expression in lung cancer tissues. Data from the *in vivo* and *in vitro* experiments demonstrated that long-term TS exposure-transformed HBE (THBE) cells acquired lung CSC-like properties. Furthermore, ΔNp63α transcriptionally activated the Notch signaling pathway to promote the acquisition of CSC-like properties by the THBE cells. TS upregulated IL-6, which increased ΔNp63α expression in THBE sphere-forming cells. Finally, SFN inhibited the TS-induced CSC-like properties of THBE cells *via* the IL-6/ΔNp63α/Notch axis.

**Conclusion**: Our data suggest that the IL-6/ΔNp63α/Notch axis plays an important role in the long-term TS exposure-induced acquisition of lung CSC-like properties and SFN intervention.

## Introduction

Lung cancer is the leading cause of cancer- related mortality worldwide. Tobacco smoke (TS) is the most important risk factor for lung cancer and accounts for 87 % of lung cancer deaths 1. Previous studies have shown that TS induces proliferation, epithelial-mesenchymal transition, invasion, and metastasis of lung cancer cells. Additionally, TS has been shown to positively affect lung cancer stem cells (CSCs) 2-4, a small population of lung cancer cells that exhibit self-renewal, CSC marker expression, and high tumorigenicity 5, 6. Therefore, determining the molecular mechanisms by which TS promotes lung CSCs is key for understanding lung carcinogenesis.

Increasing evidence has shown that several transcription factors, including transformation-related protein 63 (TP 63), are key regulators of tumor initiation and progression. TP63 encodes multiple isoforms *via* a combination of differential promoter usage and alternative C-terminal splicing. TAp63 isoforms contain full-length N-terminal transactivating domains, whilst ΔNp63 isoforms have a truncated N-terminus. Previous studies have shown that ΔNp63α is the most abundant isoform expressed in the majority of epithelial tissues and that it drives tumor formation and CSC properties 7-10. ΔNp63 is a highly specific marker in lung cancer and its upregulation has been shown to promote lung cancer migration 11, 12. Although Ratovitski previously reported that TS increased the expression of ΔNp63α in lung cancer cells 13, the role of ΔNp63α in the TS-induced acquisition of lung CSCs remains largely unexplored.

The ability of ΔNp63α to regulate tumor initiation has been linked to its effects on several signaling pathways, such as the Notch pathway which is highly conserved and plays a critical role in CSCs. Upon activation, Notch undergoes a series of proteolytic cleavages that result in the release of the Notch intracellular domain (NICD), which then translocates to the nucleus and stimulates the expression of target genes, including Hes family BHLH transcription factor 1(Hes1). Notch pathway activation is known to be a negative prognostic factor in lung cancer cells 14. Notch and Hes1 upregulation have been associated with long-term TS exposure- induced BEP2D cells and lung cancer tissues from smokers 15; however, the mechanism by which ΔNp63α regulates Notch in the TS-induced acquisition of lung CSCs remains unknown.

TS can stimulate the expression and release of inflammatory cytokines including interleukin-6 (IL-6) 16, 17, which is involved in CSC formation and the maintenance of stemness properties 18, 19. IL-6 triggers mammosphere formation and CSC self-renewal in breast cancer cells 20 and has also been demonstrated to increase lung CSC populations 21. Previously, Nelson *et al.* reported that IL-6 promoted the expression of the p63 isomer in keratinocytes during regeneration 22; however, the precise link between IL-6 and ΔNp63α in long-term TS-induced lung CSC-like properties has not yet been described.

Sulforaphane (SFN) is a potent chemopreventative compound found in vegetables of the *Brassica* genus; numerous studies have shown that SFN can target CSCs. Our previous studies showed that SFN can inhibit gastric and lung CSCs *via* Sonic hedgehog and the miR-19/GSK3β/β-catenin axis, respectively 23, 24. However, the suppression of long-term TS-induced lung CSC-like properties by SFN remains to be determined. In this study, we investigated the role of the IL-6/ΔNp63α/Notch axis in long-term TS exposure-induced acquisition of lung CSC-like properties and SFN modulation.

## Methods

### Patient sample collection

A total of 24 lung cancer tissues were collected from lung cancer patients who had undergone surgical lung cancer resection at Huai'an First People's Hospital Affiliated with Nanjing Medical University. Twelve subjects were smokers and had a cigarette smoking status of >600 cigarettes per year 25, whilst the other subjects had never smoked (n = 12). Tissues were immediately frozen in liquid nitrogen and stored at -80 °C until further analysis. All tissue sections were evaluated by an experienced pathologist to confirm the diagnosis of non-small cell lung cancer (NSCLC) according to the World Health Organization classification. All procedures involving human tumors were approved by the Ethics Committee of Nanjing Medical University (ethical clearance application number: 2016-318).

### Cell culture and preparation of cigarette smoke extract

Immortalized human bronchial epithelial (HBE) cells retain the features of the parent HBE cells 26-28 and are commonly used to study multistage bronchial epithelial carcinogenesis 29-31. Immortalized HBE cells were purchased from the Xiang Ya Central Experiment Laboratory (Changsha, China) and A549 human NSCLC cells were purchased from the Shanghai Institute of Cell Biology, Chinese Academy of Sciences (Shanghai, China). HBE and A549 cells were maintained in RPMI 1640 medium containing 10 % fetal bovine serum (Gibco, Grand Island, NY, USA), 100 U/mL of penicillin, and 100 μg/mL of streptomycin (Gibco), and incubated in 5 % CO_2_ at 37 °C.

Cigarette smoke extract (CSE) was prepared daily immediately before use, as described previously 3. Briefly, one filterless 3R4F Research Reference Cigarette (University of Kentucky, USA, 9 mg tar and 0.76 mg nicotine/cigarette) was combusted and the mainstream smoke was continuously drawn through a glass syringe containing 10 ml of pre-warmed (37 °C) fetal bovine serum-free RPMI 1640 medium at a rate of 5 min/cigarette. The resulting suspension was adjusted to pH 7.4 and passed through a filter with a 0.22 μm pore size. The solution was then referred to as a 100 % CSE solution and was diluted to the desired concentration using culture medium. A control solution was prepared using the same protocol, except that the cigarette was unlit. To mimic chronic exposure, 1 × 10^6^ HBE cells were seeded into 10 cm dishes and exposed to 0 or 2 % CSE. The medium was changed daily. This process was continued for approximately 25 weeks (55 passages). CSE-transformed HBE (THBE) cells were produced by exposing the HBE cells to 2 % CSE for 55 passages. CHBE cells were HBE cells cultured under the same conditions for 55 passages, but not exposed to CSE.

### Tumorsphere formation assay

Tumorspheres were generated by seeding THBE and CHBE cells into a 24-well ultralow plate at a density of 5000 cells/well with tumorsphere culture medium (DMEM/F12) (# 12400, Gibco) for 7 days. The tumorsphere culture medium was serum-free medium (SFM) supplemented with 4 μg/mL of insulin, 10 ng/mL of basic fibroblast growth factor, and 20 ng/mL of human recombinant epidermal growth factor. Representative field images were then acquired by microscopy (original magnification, 400×) and the number of tumorspheres with a diameter >100 μm was counted. Three independent experiments were performed.

### Colony formation assay

Approximately 500 CHBE, THBE, and A549 cells were seeded into 6-well plates and cultured at 37 °C for 10 days to allow colony formation. Cells were subsequently washed in phosphate-buffered saline (PBS), fixed in 4 % paraformaldehyde, and stained with 0.1 % crystal violet (Sigma-Aldrich, St. Louis, MO, USA). Images were captured using a fluorescence microscope, and the number of colonies (>50 cells/colony) was determined. Three independent experiments were performed.

### Western blot analyses

Cells and tissues were harvested after the indicated treatments and lysed in RIPA buffer containing 1× protease inhibitor cocktail (Pierce, Rockford, IL, USA) and EDTA. A Bradford Protein Assay Kit was used to quantify the levels of protein in the total protein lysates. Equal amounts of total protein (60 μg) were separated by 10 % sodium dodecyl sulfate-polyacrylamide gel electrophoresis and transferred to polyvinylidene fluoride membranes (Bio-Rad, Hercules, CA, USA) for immunoblotting. The membranes were blocked with 5 % nonfat dry milk at 25 °C for 1 h on a rotary shaker and incubated overnight at 4 °C with the following primary antibodies: ΔNp63α (TA327976; OriGene Technologies, Rockville, MD, USA); CD133 (18470-1-AP), ALDH1A1 (15910-1-AP), Nanog (14295-1-AP), Oct4 (60242-1-Ig), Sox2 (11064-1-AP), ZO-1 (21773-1-AP), E-cadherin (20874-1-AP), Vimentin (10366-1-AP), N-cadherin (22018-1-AP), IL-6 (21865-1-AP), and β-tubulin (10094-1-AP; Proteintech, Rosemont, IL, USA); NICD (ab83232) and Hes1 (ab119776; Abcam, Cambridge, Massachusetts, US); β-actin (AP0060; Biogot Technology, Nanjing, China). The membranes were then washed with Tris-buffered saline/Tween and incubated with secondary antibodies. For densitometric analyses, protein bands on the blots were normalized to β-actin or β-tubulin with Eagle Eye II software. Three independent experiments were performed.

### Transient transfection

THBE cells were plated onto 6-well plates at a density of approximately 2 × 10^5^ cells in RPMI 1640 medium containing 10 % fetal bovine serum without antibiotics. After incubation for 12 h, the cells were transiently transfected with pcMV-ΔNp63α plasmids (2 μg) or a control vector (2 µg) along with ΔNp63α- siRNA (75 nM) or control-siRNA (75 nM) and Lipofectamine 2000 reagent (Invitrogen, Carlsbad, CA, USA), according to the manufacturer's instructions. Twelve hours later the cells were trypsinized and incubated in serum-free medium for another 4 days. pcMV-ΔNp63α (RG225987) was purchased from OriGene Technologies, whilst ΔNp63α-specific siRNAs 32 and the control siRNA were synthesized by Invitrogen.

### Immunofluorescent staining

For immunofluorescent staining, the cells were seeded into a 24-well ultralow plate containing tumorsphere culture medium, fixed in 4 % paraformaldehyde, and permeabilized with 0.5 % Triton X-100 for 15 min. The slides were then incubated overnight with primary antibodies (CD133, ALDH1A1, Nanog, NICD, and Hes1) in a blocking solution at 4 °C in a humidified chamber. The slides were washed three times with PBS and incubated with secondary antibodies for 1 h at 25 °C in a humidified chamber. Finally, cells were incubated with 4'6-diamidino-2-phenylindole (Sigma-Aldrich) for 15 min and images were obtained by confocal microscopy.

### Dual-luciferase reporter assay

Wt-Notch1 and mut-Notch1 promoters were each cloned into the pGL3 vector by directional cloning. THBE cells were co-transfected with ΔNp63α plasmids, ΔNp63α-siRNA, 0.2 μg of firefly luciferase reporter vector, and 0.02 μg of control vector containing *Renilla* luciferase pRL-SV40, using lip 2000 (Invitrogen) in 24-well plates. Luciferase assays were performed 48 h after transfection, with firefly luciferase activity normalized to *Renilla* luciferase activity. Luciferase reporter gene plasmids containing the wt-Notch1 and mut-Notch1 promoters were obtained from GENEray (Shanghai, China) and pRL-SV40 plasmids were obtained from Promega (Madison, WI, USA).

### Enzyme linked immunosorbent assay (ELISA)

Cells from lung cancer tissues were lysed using RIPA buffer and their IL-6 levels were quantified using an ELISA kit in accordance with the manufacturer's instructions (Nanjing JianCheng Bioengineering Institute, Nanjing, China).

### Cell treatment with DAPT, SFN and IL-6

To determine the effects of DAPT (#HY-13027; purity 99.97 %; Med Chem Express, Monmouth Junction, NJ, USA), SFN (#13755; purity 98.9 %; Cayman Chemical, Ann Arbor, MI, USA), and human recombinant IL-6 (# 206-IL; purity > 97 %; R&D Systems, Minneapolis, MN, USA) on THBE lung CSC-like properties, different concentrations of DAPT (0, 20, and 40 μM), SFN (0, 5, 10, and 15 μM), and IL-6 (0, 10, and 20 ng/mL) were added to the tumorsphere culture medium with 0.1 % dimethyl sulfoxide (DMSO) (Sigma) or PBS as the vehicle control. For IL-6 and SFN co-treatment, THBE sphere-forming cells were treated with SFN (15 μM) for 3 days and then with IL-6 (20 ng/mL) for 24 h.

### Cell viability assay

HBE and CHBE cells were seeded into 96-well culture plates at a density of 2000 cells/well, cultured in serum-supplemented medium, and treated with various SFN concentrations for 7 days. Cell viability was assessed using an Enhanced Cell Counting Kit-8 (Beyotime, Shanghai, China).

### *In vivo* xenograft and treatment experiments

Male BALB/c nude mice (specific pathogen-free grade; 4-5 weeks-old) were purchased from the Animal Research Center of Nanjing Medical University. The mice were handled in accordance with the guidelines of the Animal Care and Welfare Committee of Nanjing Medical University (IACUC- 1802010).

To evaluate the effect of THBE cells on tumor growth, 15 mice were randomly divided into three groups of five mice. Next, 1 × 10^6^ exponentially growing THBE cells, CHBE cells, and A549 cells were suspended in 100 μL of PBS and subcutaneously injected into the front dorsum of the nude mice. Tumor volume was measured every 2-3 days for 2 weeks using an electronic caliper. At the scheduled time of surgery, the mice were sacrificed and their tumors were removed and weighed.

To investigate whether SFN affected the growth of tumor cells, 1 × 10^6^ THBE cells were subcutaneously injected into the front dorsum of the nude mice. Approximately 10 days later, when palpable tumors were present, the mice were randomly administered either SFN (25 or 50 mg/kg/body weight) or the vehicle control (n = 5 per group). Every three days, the treatments were repeated and the body weight of the mice was measured. After 3 weeks of treatment, the mice were sacrificed and their tumors were removed, photographed, measured, frozen, and stored at -80 °C until further analysis.

### Statistical analysis

Statistical analyses were performed with SPSS 25.0 software (SPSS, Chicago, IL, USA). All data were expressed as the mean ± standard deviation. One-way analysis of variance (ANOVA) was used to compare differences among multiple groups, whilst unpaired two-tailed Student's *t* tests were used to compare differences between two groups. Pearson's correlation coefficient (*r*) was used to analyze the relationship between ΔNp63α protein expression and CD133, Oct4, and IL-6 levels. *P* values < 0.05 were considered to indicate significant differences.

## Results

### ΔNp63α upregulation correlates with TS-associated lung CSC-like properties

**Figure 1:**
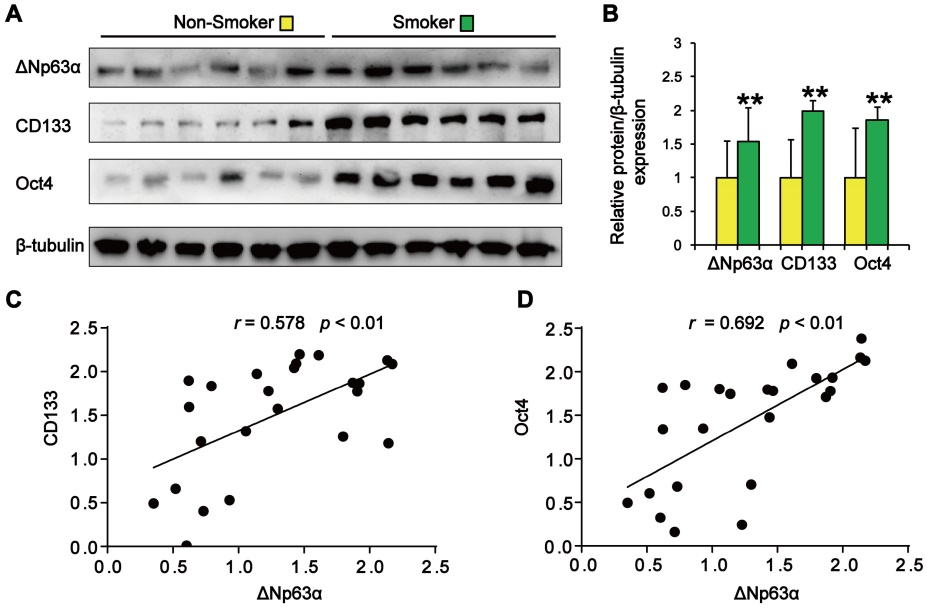
** ΔNp63α upregulation correlates with TS-associated lung CSC-like properties. (A)** Western blot analysis of ΔNp63α, CD133, and Oct4 protein expression in lung cancer tissues. **(B)** Densitometry analysis (fold-change) of western blot after β-tubulin normalization (n = 12 per group). **(C and D)** The Pearson correlation coefficient (*r*) was used to analyze the relationship between ΔNp63α protein expression and the levels of CD133 **(C)** and Oct4 **(D)** in lung cancer specimens (n = 24). Data are expressed as the mean ± SD. Significance was assessed by unpaired two-tailed Student's* t* tests. ** *P* < 0.01 compared to the non-smoker group.

We initially evaluated the correlation between ΔNp63α expression and the smoking status of patients with lung cancer. As shown in Figure 1A and B, tumor samples from smokers showed significantly higher levels of ΔNp63α than samples from subjects who had never smoked. Western blot analysis of lung CSC markers revealed that CD133 and Oct4 expression levels were significantly higher in the lung cancer tissues of smokers (Figure 1A and B). Furthermore, ΔNp63α expression levels positively correlated with the expression of CD133 (*r* = 0.578, *p* < 0.01) and Oct4 (*r* = 0.692, *p* < 0.01) in lung cancer tissues (Figure 1C and D). These results suggest that ΔNp63α expression is correlated with TS-induced lung CSC-like properties.

### Long-term TS exposure induces lung CSC-like phenotype acquisition

**Figure 2:**
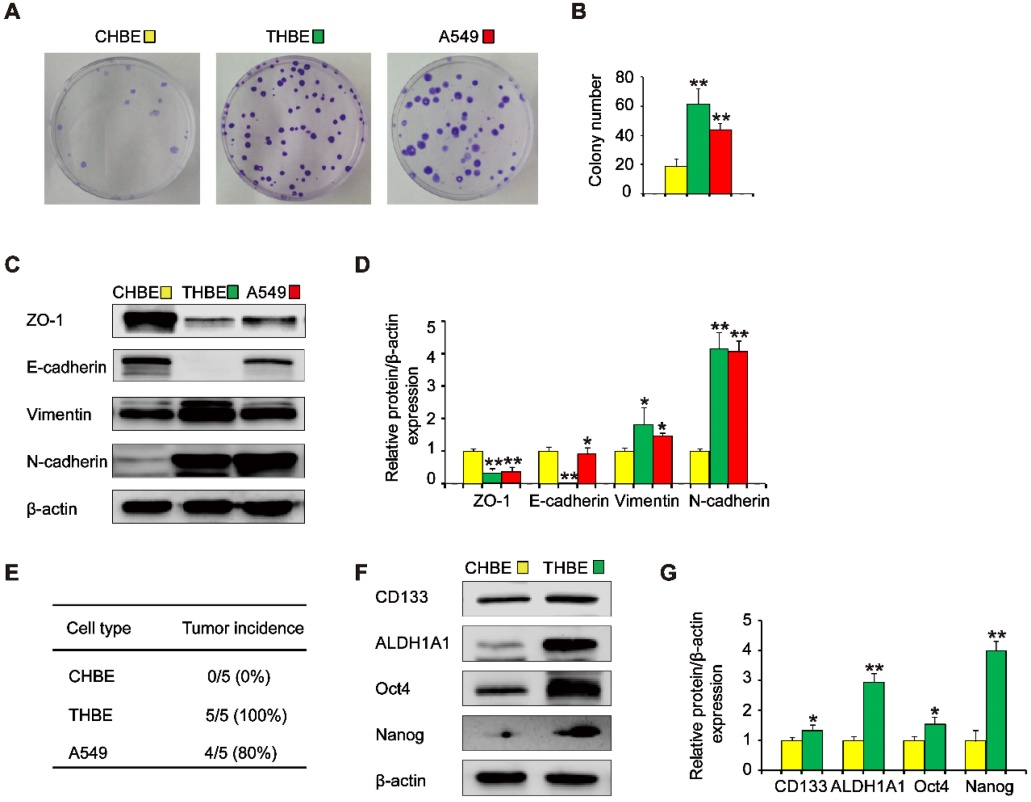
** Long-term TS exposure-induced acquisition of the CSC-like phenotype.** HBE cells were exposed to 2 % CSE (cigarette smoke extract) for 55 passages. Images of cell colonies **(A)** were taken and the number of colonies **(B)** was counted. **(C)** ZO-1, E-cadherin, Vimentin, and N-cadherin levels in TS-treated HBE cells were determined using A549 cells as positive controls. **(D)** Densitometric analyses of western blots of ZO-1, E-cadherin, Vimentin, and N-cadherin were performed following β-actin normalization. **(E)** 1 × 10^6^ non-CSE exposed control HBE cells, TS-exposed HBE cells, and A549 cells were subcutaneously injected in the front dorsum of nude mice and tumor incidence was analyzed 2 weeks later. **(F)** CHBE and TS-treated HBE cells were cultured in serum-free medium (SFM) for 7 days. Western blot analysis of lung CSC markers was then performed. **(G)** Densitometric analyses of western blots of CD133, ALDH1A1, Oct4, and Nanog were measured after β-actin normalization. Three independent experiments were performed. Data are expressed as the mean ± SD. Significance was assessed by one-way ANOVA or unpaired two-tailed Student's* t* tests. * *P* < 0.05, ** *P* < 0.01 compared to CHBE cells. THBE: HBE cells exposed to CSE for 55 passages; CHBE: HBE cells cultured under the same conditions for 55 passages, but not exposed to CSE. A549 cells were used as positive controls.

To investigate the effects of ΔNp63α on TS-induced lung CSC-like properties, we used CSE to induce the transformation of HBE cells. To determine whether cells chronically exposed to CSE acquired colony formation abilities, their colony formation was evaluated. Colony formation was significantly higher in cells exposed to CSE over a long period of time than in non-CSE exposed control cells (Figure 2A and B). Next, we examined the expression of epithelial and mesenchymal markers in CSE-treated cells. The expression levels of the epithelial markers ZO-1 and E-cadherin were decreased, whilst Vimentin and N-cadherin levels were increased in CSE-exposed cells (Figure 2C and D). Additionally, tumor incidence was 100 % (5/5) in the group of mice injected with CSE-transformed HBE cells, yet was 80 % (4/5) for those injected with A549 cells and 0 % (0/5) for those injected with non-CSE-exposed control cells (Figure 2E). Moreover, CSE-transformed HBE cells cultured with growth factors expressed significantly higher levels of lung CSC markers, including CD133, ALDH1A1, Oct4, and Nanog (Figure 2F and G), indicating that THBE sphere-forming cells exhibited lung CSC-like features. These results suggest that long-term TS exposure induced the acquisition of a CSC-like phenotype.

### ΔNp63α promotes the TS-induced acquisition of CSC-like properties in HBE cells

**Figure 3:**
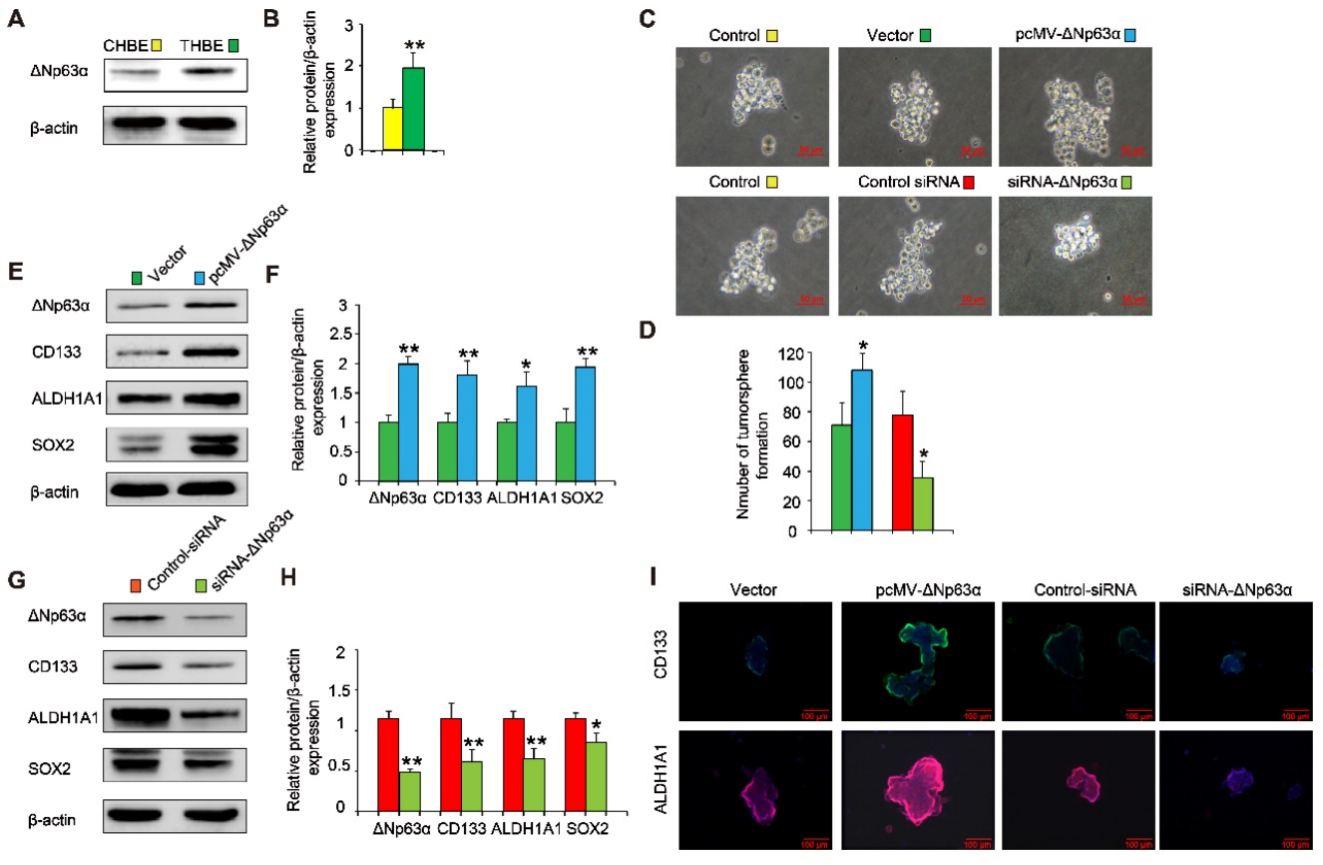
** ΔNp63α promotes the TS-induced acquisition of CSC-like properties in HBE cells. (A)** ΔNp63α expression was measured in CHBE and THBE sphere-forming cells. **(B)** Densitometric analyses of western blots for ΔNp63α were conducted after β-actin normalization. THBE sphere-forming cells were transfected with a control vector, ΔNp63α plasmids, control-siRNA, or ΔNp63α-siRNA for 4 days. Representative tumorsphere images were acquired **(C)** (original magnification, 400×) and the number of tumorspheres was quantified **(D)**. **(E)** Western blotting of ΔNp63α, CD133, ALDH1A1, and Sox2 was performed, and **(F)** the relative protein levels were determined in the presence or absence of ΔNp63α plasmid transfection. THBE sphere-forming cells were treated with or without ΔNp63α-siRNA for 4 days and the expression of the indicated proteins was detected by western blot analyses **(G)**. **(H)** Densitometric analyses of western blots of ΔNp63α, CD133, ALDH1A1, and Sox2 were performed after β-actin normalization. **(I)** Immunofluorescence staining of CD133 and ALDH1A1 in THBE sphere-forming cells for the indicated groups. Three independent experiments were performed; original magnification, 200×. Data are expressed as the mean ± SD. Significance was assessed by unpaired two-tailed Student's* t* tests. * *P* < 0.05, ** *P* < 0.01 compared to the vector or control-siRNA group.

Since elevated ΔNp63α expression was positively correlated with levels of lung CSC markers in lung cancer tissues and chronic TS exposure induced the upregulation of ΔNp63α (Figure 3A and B), we investigated whether ΔNp63α plays a role in TS-induced CSC-like properties. ΔNp63α plasmids were transfected into CSE-transformed HBE cells and cultured in SFM medium for 4 days; we then analyzed the tumorsphere formation capacity of these cells. Exogenous ΔNp63α induced larger tumorspheres compared to those formed by control cells (Figure 3C); the number of tumorspheres was also increased following transfection with ΔNp63α plasmids (Figure 3D). Moreover, the forced expression of ΔNp63α significantly increased the protein levels of lung CSC markers including CD133, ALDH1A1, and Sox2 (Figure 3E and F). Immunofluorescence images confirmed that ΔNp63α increased the expression of CD133 and ALDH1A1 in the THBE sphere-forming cells compared to the vector control cells (Figure 3I). In contrast, using siRNA to down-regulate ΔNp63α expression reduced the number and size of the tumorspheres and suppressed the expression of lung CSC markers in THBE sphere-forming cells (Figure 3). These data indicate that ΔNp63α is important in the TS-induced acquisition of lung CSC-like properties.

### Notch is involved in the TS-induced acquisition of lung CSC-like properties

**Figure 4:**
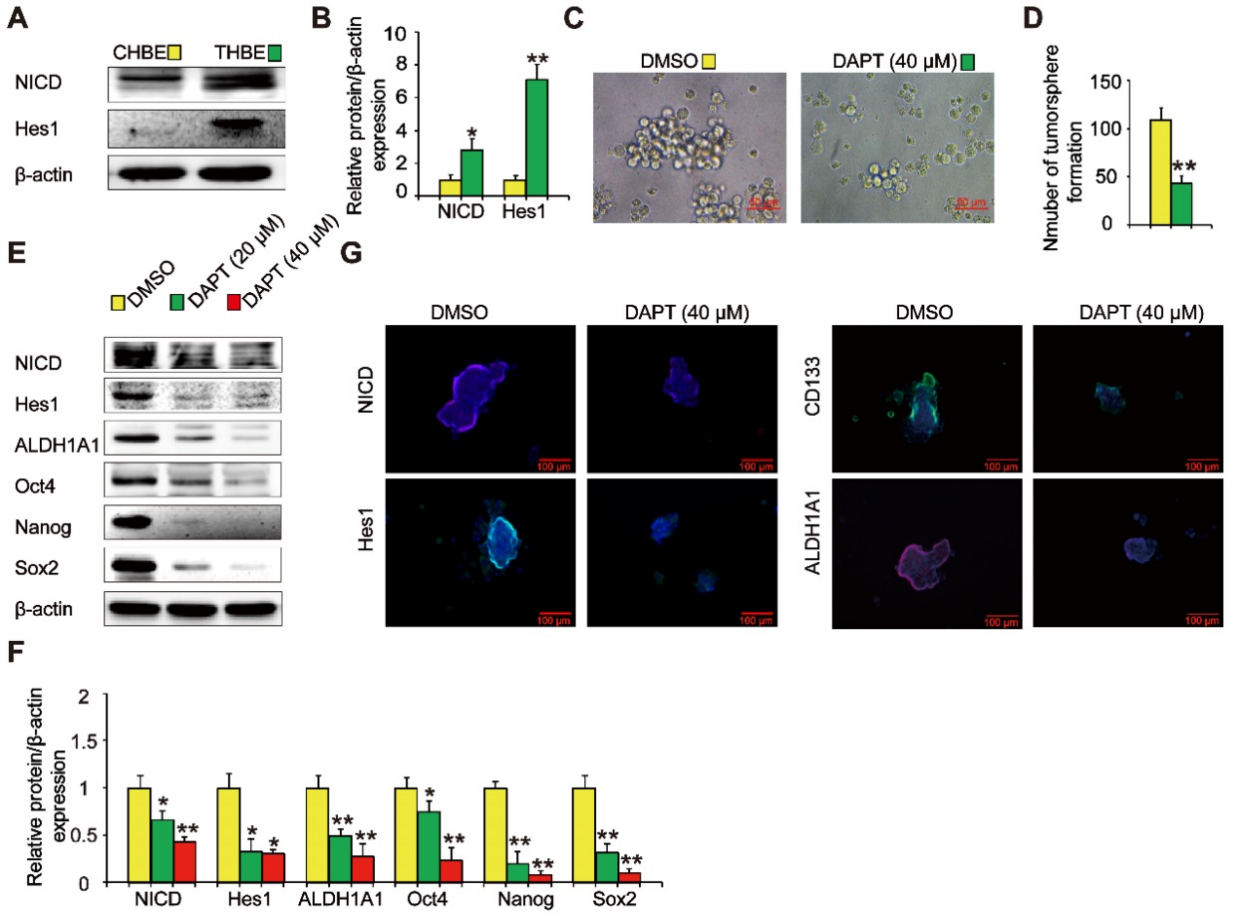
** Notch is involved in the TS-induced acquisition of lung CSC-like properties. (A)** The expression of Notch pathway proteins, including NICD and Hes1, was detected by western blotting. **(B)** Densitometric analyses of the western blots of NICD and Hes1 were performed after β-actin normalization. THBE sphere-forming cells were treated with DAPT for 4 days. Representative tumorsphere images (original magnification, 400×) were acquired **(C)** and tumorspheres were quantified **(D)**. **(E)** Western blotting was used to detect the expression of lung CSC markers. **(F)** The indicated protein levels relative to β-actin were assessed by densitometric analysis. **(G)** Immunofluorescence staining of NICD, Hes1, CD133, and ALDH1A1 in THBE sphere-forming cells for the indicated groups, original magnification, 200×. Three independent experiments were performed. Data are expressed as the mean ± SD. Significance was assessed by unpaired two-tailed Student's* t* tests or one-way ANOVA. * *P* < 0.05, ** *P* < 0.01 compared to the control group.

The Notch signaling pathway is crucial in the maintenance of CSC traits. We found that NICD and Hes1 levels were upregulated in THBE sphere- forming cells (Figure 4A and B), indicating Notch pathway activation. To examine the role of the Notch pathway in THBE sphere-forming cells, we used DAPT, a Notch pathway inhibitor, demonstrating that the size and number of tumorspheres were significantly reduced following DAPT treatment (Figure 4C and D). Western blotting showed that the levels of ALDH1A1, Oct4, Nanog, and Sox2 proteins were also markedly reduced by DAPT (Figure 4E and F), with similar alterations observed for the lung CSC markers by immunofluorescence analysis (Figure 4G). These results suggest that the Notch pathway modulates the activity of THBE lung CSCs.

### ΔNp63α transcriptionally regulates the effect of the Notch pathway on TS-induced lung CSC-like properties

**Figure 5:**
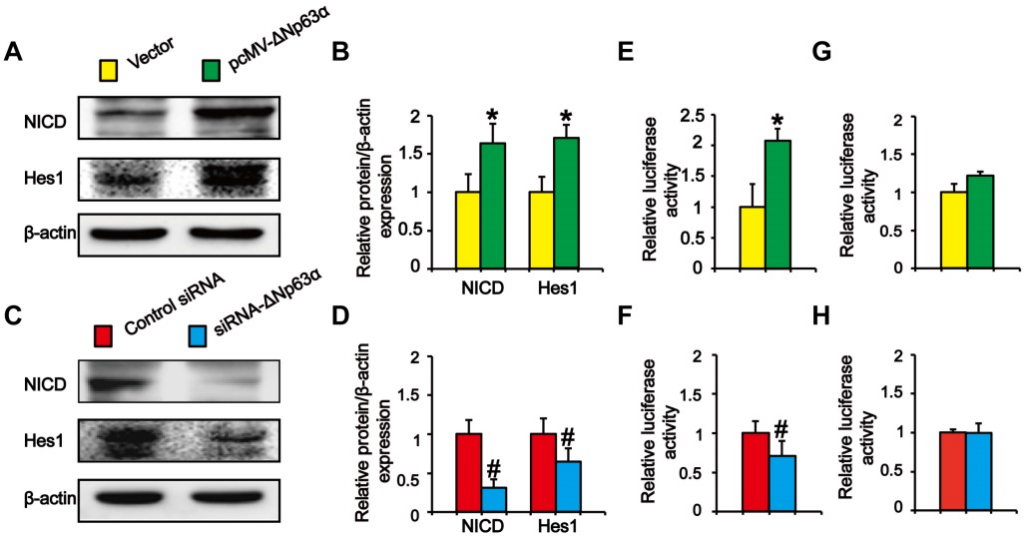
** ΔNp63α transcriptionally regulates the effect of the Notch pathway on TS-induced lung CSC-like properties. (A and C)** THBE sphere-forming cells were transfected with ΔNp63α plasmids, a control vector, siRNA-ΔNp63α, or control- siRNA for 4 days and the protein levels of NICD and Hes1 were determined by western blotting. **(B and D)** Densitometry results are shown as the fold-change compared to the vector or control siRNA after β-actin normalization. **(E and F)** THBE sphere-forming cells were co-transfected with wt-Notch1 promoter-luciferase, ΔNp63α **(E)**, or ΔNp63α-siRNA **(F)**, and luciferase activity was measured after incubating for 3 days.** (G and H)** THBE sphere-forming cells were co-transfected with mut-Notch1 promoter-luciferase, ΔNp63α **(G)**, or ΔNp63α siRNA **(H)**, and their luciferase activity was measured. Three independent experiments were performed. Data are expressed as the mean ± SD. Significance was assessed by unpaired two-tailed Student's* t* tests. * *P* < 0.05, compared to the vector group. # *P* < 0.05, compared to the control siRNA group.

Previous experiments showed that ΔNp63α binds to the Notch1 promoter and directly activates Notch in MCF-7 cells 20. To explore whether ΔNp63α triggered Notch to promote the formation of THBE sphere-forming cells, we transfected ΔNp63α plasmids into CSE-transformed cells and cultured the cells in SFM medium. Western blot analysis indicated that NICD and Hes1 levels were significantly higher in ΔNp63α cells than in empty vector cells (Figure 5A and B), whilst inhibiting ΔNp63α reduced the levels of Notch-related pathway proteins (Figure 5C and D). To determine whether ΔNp63α regulated Notch at the transcriptional level, the ΔNp63α fragment containing the Notch1 promoter response element was cloned into the PGL3 plasmid vector and a dual-luciferase reporter assay was conducted to detect the transcriptional activation of Notch1 in response to ΔNp63α. Luciferase activity was enhanced following ΔNp63α transfection, whereas siRNA-ΔNp63α reduced its activity (Figure 5E and F). Furthermore, after mutating the Notch1 promoter binding site for ΔNp63α, ΔNp63α overexpression or knockdown did not affect the fluorescence intensity of the mutated Notch1 promoter (Figure 5G and H). Our data suggest that ΔNp63α triggered the transcriptional activation of Notch during the TS-induced acquisition of CSC-like properties.

### IL-6 increases ΔNp63α levels in TS exposure-induced lung CSC-like properties

**Figure 6:**
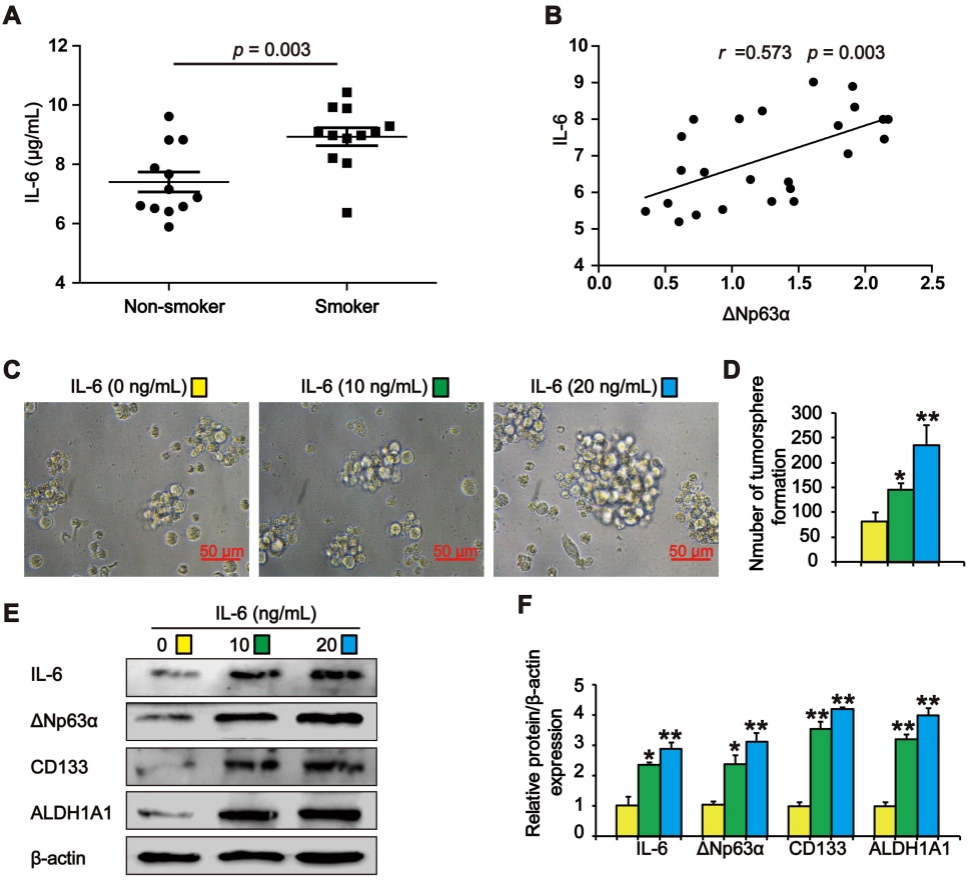
** IL-6 increases ΔNp63α in cells with TS exposure-induced lung CSC-like properties. (A)** IL-6 levels in the lung cancer tissues of smokers and non-smokers were analyzed by ELISA (n = 12 per group). **(B)** IL-6 expression was significantly and positively correlated with ΔNp63α expression in the lung cancer specimens (Pearson correlation coefficient analysis). **(C-F)** THBE sphere-forming cells were exposed to various IL-6 concentrations for 7 days. The size (original magnification, 400×) **(C)**, and number **(D)** of tumorspheres were measured. The expression levels of ΔNp63α and lung CSCs markers were measured by western blotting **(E)** and densitometric analyses **(F).** Three independent experiments were performed. Data are expressed as the mean ± SD. Significance was assessed by unpaired two-tailed student's *t* tests or one-way ANOVA test. * *P* < 0.05, ** *P* < 0.01, compared to the control group.

Previous studies have shown that TS stimulates IL-6 expression to promote tumor initiation. We observed higher IL-6 levels in the lung cancer tissues of smokers than in those of subjects who had never smoked (Figure 6A). Pearson correlation analysis showed that ΔNp63α was positively correlated with IL-6 levels in lung cancer tissues (Figure 6B). To investigate whether TS-induced IL-6 promoted the acquisition of lung CSC-like properties, CSE-transformed HBE cells were treated with various IL-6 concentrations for 7 days and tumorsphere formation was detected. As shown in Figure 6C and D, the size and number of tumorspheres was significantly increased by IL-6. Moreover, IL-6 increased the expression of ΔNp63α and lung CSC markers including CD133 and ALDH1A1 (Figure 6E and F). Collectively, these data suggest that IL-6 stimulated ΔNp63α activation during the TS-induced acquisition of lung CSC-like properties.

### SFN suppresses long-term TS exposure-induced lung CSC-like properties

**Figure 7:**
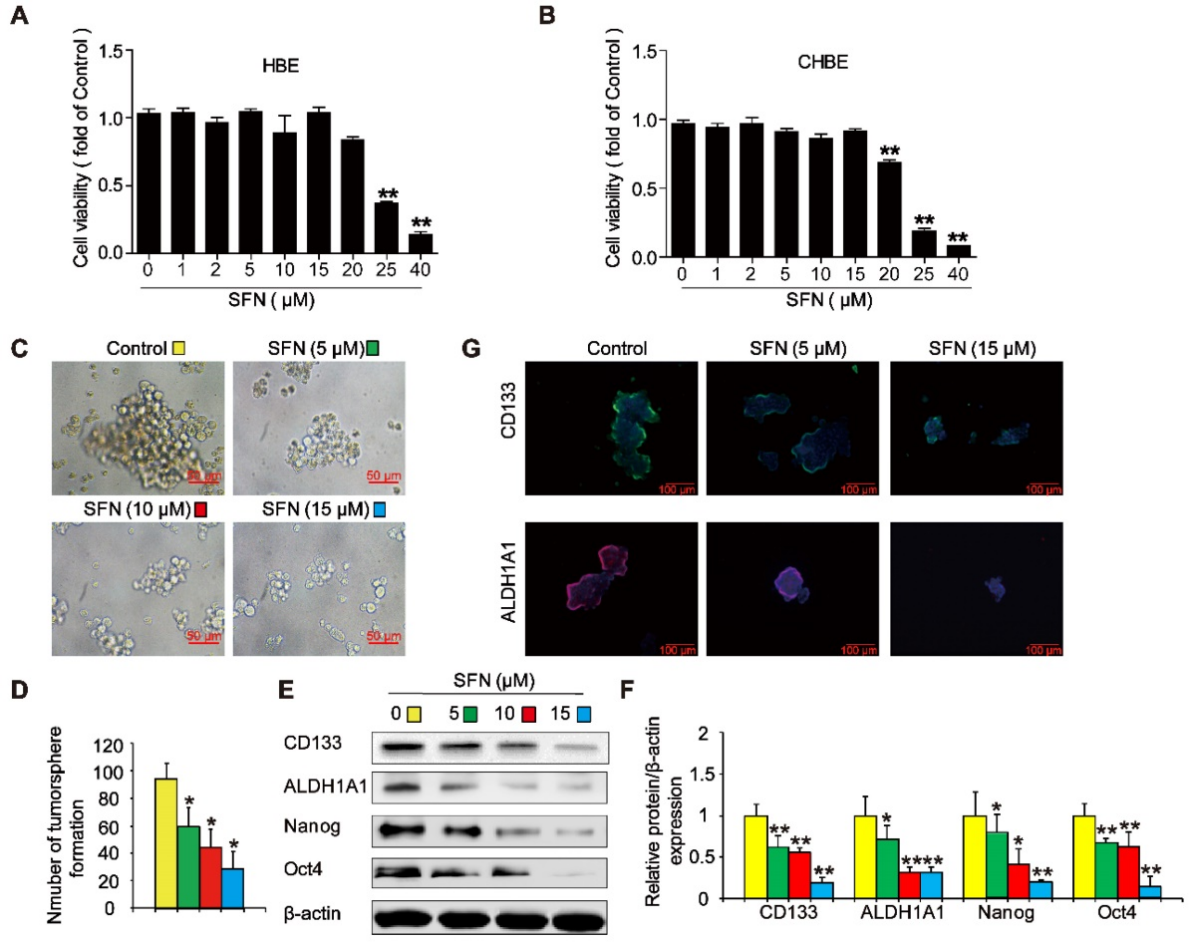
** SFN suppresses long-term TS exposure-induced lung CSC-like properties. (A and B)** Effect of SFN on the viability of HBE cells and CHBE cells. Cells were treated with various SFN concentrations for 7 days and cell viability was measured using a CCK8 assay in HBE cells **(A)** and CHBE cells **(B)**. THBE sphere-forming cells were treated with different SFN concentrations (0, 5, 10, and 15 µM) for 7 days. Representative tumorsphere images (original magnification, 400×) were taken **(C)**, and tumorspheres were quantified **(D)**. **(E)** The expression of lung CSC markers was determined by western blotting. **(F)** The levels of the indicated proteins relative to β-actin were assessed by densitometric analysis. **(G)** Immunofluorescence staining of CD133 and ALDH1A1 in THBE sphere-forming cells for the indicated groups; original magnification, 200×. Three independent experiments were performed. Data are expressed as the mean ± SD. Significance was assessed by one-way ANOVA. * *P* < 0.05, ** *P* < 0.01 compared to the control group.

To evaluate whether SFN suppressed the formation of tumorspheres in CSE-transformed HBE cells, THBE sphere-forming cells were exposed to various SFN concentrations for 7 days. The CCK8 assay revealed no apparent toxicity in HBE cells and CHBE cells treated with up to 15 µM SFN (Figure 7A and B); however, SFN inhibited tumorsphere formation in a concentration-dependent manner between 5 and 15 µM, significantly reducing both the size and number of tumorspheres with increasing SFN concentrations (Figure 7C and 7D). Moreover, the levels of CD133, ALDH1A1, Oct4, and Nanog proteins were reduced in THBE sphere- forming cells exposed to different SFN concentrations (Figure 7E and F) and SFN reduced the fluorescence intensity of CD133 and ALDH1A1 in these cells (Figure 7G). Together, these data suggest that SFN suppressed the CSC-like properties of TS-transformed HBEs.

### IL-6/ΔNp63α/Notch mediates the suppression of long-term TS exposure-induced lung CSC-like properties by SFN

**Figure 8:**
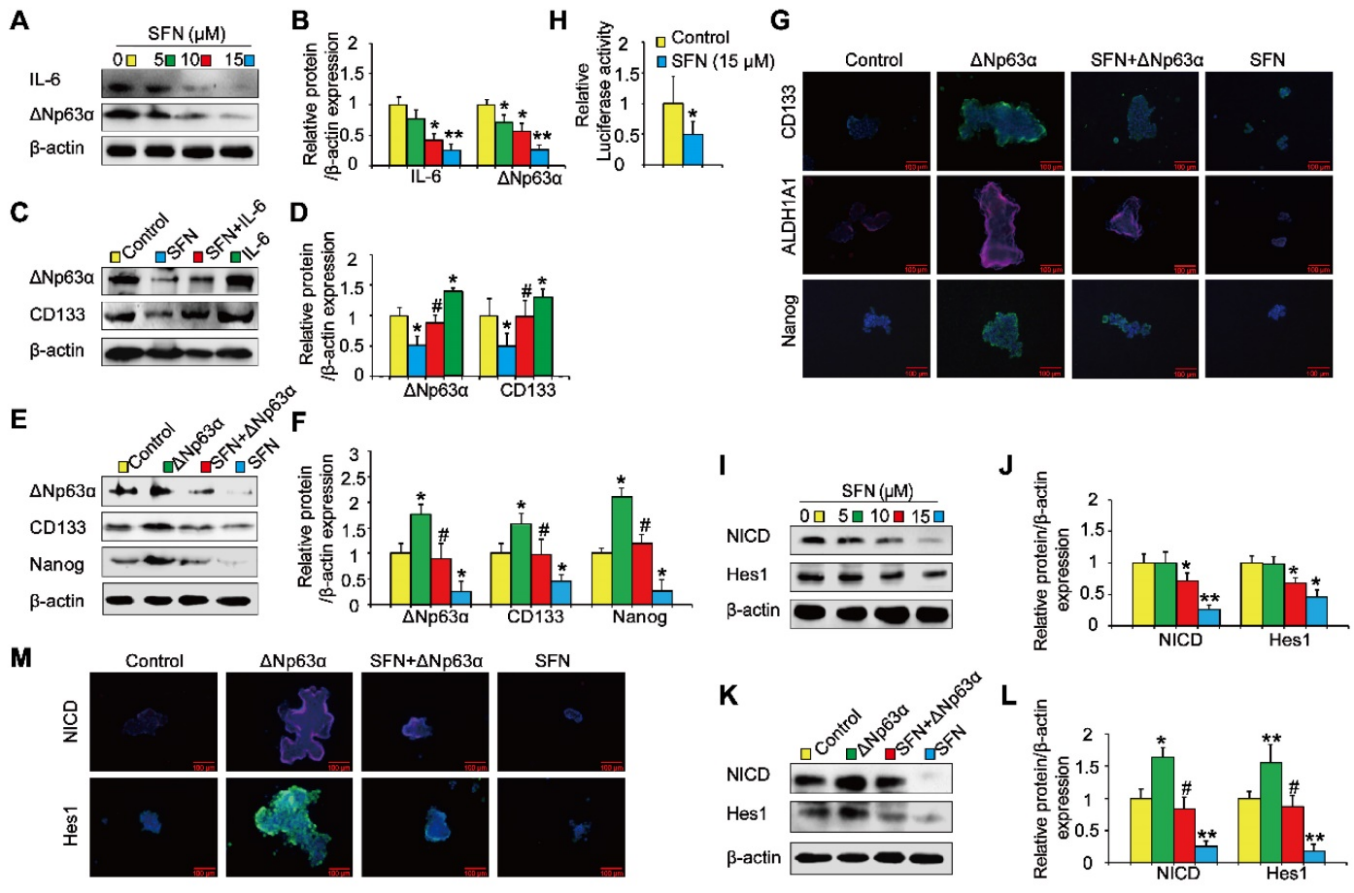
** IL-6/ΔNp63α/Notch axis mediates the suppression of long-term TS exposure-induced lung CSC-like properties by SFN. (A)** THBE tumorspheres were treated with different SFN concentrations (0, 5, 10, and 15 µM) for 7 days. The protein expression levels of IL-6 and ΔNp63α were detected by western blotting. **(B)** The levels of the indicated proteins relative to β-actin were assessed by densitometric analysis. **(C)** THBE sphere-forming cells were pretreated with SFN (15 μM) for 3 days, stimulated by IL-6 (20 ng/mL) for 24 h, then ΔNp63α and CD133 expression were detected by western blotting.** (D)** Densitometry results are shown as the fold-change compared to the control group after β-actin normalization.** (E)** Tumorspheres formed by THBE cells were transfected with ΔNp63α plasmids, cultured in the presence or absence of 15 µM SFN for 4 days, and the protein levels of ΔNp63α and lung CSCs markers were then measured by western blotting. **(F)** Densitometry results are shown as the fold-change compared to the control group after β-actin normalization.** (G)** Immunofluorescence staining of CD133, ALDH1A1, and Nanog in THBE sphere-forming cells for the indicated groups, original magnification, 200×. **(H)** The luciferase activity of the Notch1 promoter was measured in the THBE sphere-forming cells after 4 days of incubation with SFN.** (I)** Western blot analyses were performed, and **(J)** the relative protein levels of NICD and Hes1 were determined. **(K)** NICD and Hes1 expression in ΔNp63α transfected THBE sphere-forming cells with or without of SFN (15 µM) treatment. **(L)** Densitometric analyses of western blots of NICD and Hes1 were performed after β-actin normalization. **(M)** Immunofluorescence staining of NICD and Hes1 in THBE sphere-forming cells for the indicated groups, original magnification, 200×. Three independent experiments were performed. Data are expressed as the mean ± SD. Significance was assessed by one-way ANOVA or unpaired two-tailed Student's* t* tests. * *P* < 0.05, ** *P* < 0.01 compared to the control group; # *P* < 0.05 compared to the SFN treatment group.

Next, we investigated the role of the ΔNp63α/ Notch axis in the suppression of long-term TS exposure-induced lung CSC-like properties by SFN. As shown in Figures 8A and 8B, SFN treatment reduced the expression of IL-6 and ΔNp63α. To examine whether IL-6 modulated the inhibitory effects of SFN on TS-induced lung CSC-like properties, THBE sphere-forming cells were exposed to IL-6 with or without SFN treatment. The effects of SFN on ΔNp63α and CD133 were found to be attenuated by IL-6 treatment (Figure 8C and D). Transient ΔNp63α plasmid transfection was conducted to upregulate ΔNp63α expression. Western blotting assays showed that overexpressing ΔNp63α reduced the SFN-induced suppression of lung CSC markers (line 3 compared to line 4; Figure 8E and F). Furthermore, dual-luciferase reporter assays revealed that SFN inhibited the luciferase activity of Notch (Figure 8H), suggesting that SFN suppresses the Notch pathway activation. As expected, SFN reduced NICD and Hes1 protein expression in THBE sphere- forming cells in a concentration-dependent manner (Figure 8I and J), yet these effects were attenuated by the transfection of ΔNp63α plasmids in these cells (line 3 compared to line 4; Figure 8K, L, and M).

**Figure 9:**
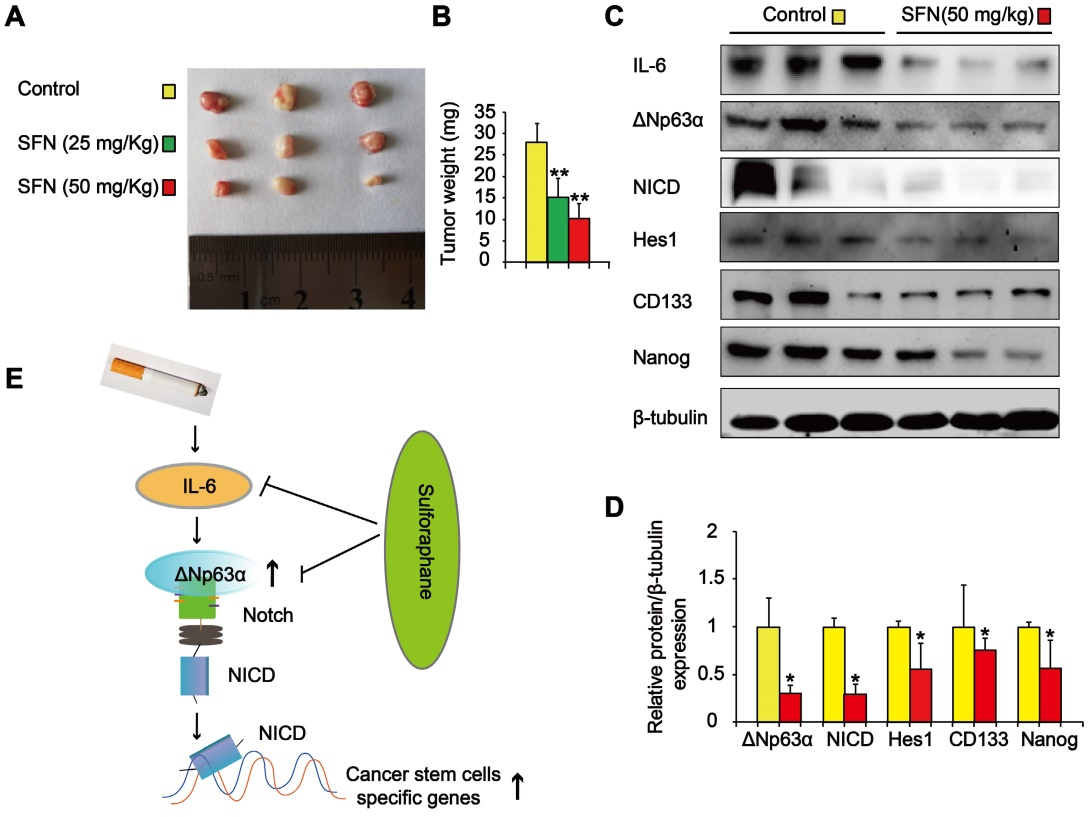
** SFN suppresses the IL-6/ΔNp63α/Notch axis to inhibit TS-induced lung CSC-like properties.** THBE cells (1 × 10^6^) were injected subcutaneously into the flank of nude mice. Approximately 10 days later, when palpable tumors were present, the mice were randomly administered either SFN (25 or 50 mg/kg body weight) or a vehicle control for 3 weeks, then tumor size **(A)** and weight **(B)** were measured. **(C)** Western blotting and **(D)** the relative protein levels of IL-6, ΔNp63α, NICD, Hes1, and lung CSC markers were determined. Five animal samples per group were used for densitometric analysis. **(E)** Schematic diagram showing the mechanism involved in the TS-mediated induction of lung CSCs. Exposure of human bronchial epithelial HBE cells to CSE induces the acquisition of lung CSC-like properties by activating ΔNp63α/Notch *via* IL-6 upregulation. SFN inhibits TS-triggered lung CSC-like properties *via* the IL-6/ΔNp63α/Notch axis. Data are expressed as the mean ± SD. Significance was assessed by one-way ANOVA or unpaired two-tailed Student's* t* tests. * *P* < 0.05, ** *P* < 0.01 compared to the control group.

We also determined the effects of SFN on TS-mediated CSC-like properties *in vivo*. Nude mice with CSE-transformed HBE cell subcutaneous xenografts were treated with SFN (25 or 50 mg/kg body weight) or a vehicle control. SFN treatment significantly reduced tumor size and weight compared to the control group (Figure 9A and B). Moreover, SFN (50 mg/kg body weight) treatment significantly reduced the levels of IL-6, ΔNp63α, NICD, Hes1, and lung CSC markers (CD133 and Nanog) (Figure 9C and D). The *in vitro* and *in vivo* data revealed that the IL-6/ΔNp63α/Notch axis mediated the suppression of long-term TS exposure-induced lung CSC-like properties by SFN.

## Discussion

As a major risk factor for lung cancer, TS plays a critical role in tumor initiation, with the induction of CSCs by TS being an early event in carcinogenesis. ΔNp63α is a lung cancer-related oncogene; however, its molecular mechanisms in the TS exposure-induced acquisition of lung CSC properties remain unclear. This study revealed that ΔNp63α levels were increased in the lung cancer tissues of smokers and positively correlated with the levels of lung CSC markers in lung cancer tissues. Tumorsphere formation capacity, lung CSC marker expression, and *in vivo* tumor incidence revealed that long-term CSE exposure-transformed HBE cells acquired lung CSC-like properties. Moreover, TS increased IL-6 levels to induce ΔNp63α expression, which transcriptionally regulated Notch pathway activation to promote TS-triggered lung CSC-like properties. We also showed that the IL-6/ΔNp63α/Notch axis mediated the suppression of long-term TS exposure-induced lung CSC-like properties by SFN. Taken together, these data suggest that the IL-6/ΔNp63α/Notch axis plays an important role in the TS exposure-induced acquisition of lung CSC-like properties and mediates the effects of SFN.

Several cellular markers are commonly used to identify lung CSCs. CD133 affects the self-renewal capacity of sphere-forming and side-population lung cancer cells 33. Cells with high ALDH1A1 expression display CSC features, including an increased capacity for proliferation, self-renewal, and tumorigenicity 34. Oct4 is essential for maintaining the self-renewal of pluripotent embryonic stem (ES) cells 35, with a recent study showing that Oct4 plays a crucial role in maintaining CSC traits in lung cancer-derived CD133-positive cells 36. Nanog, a key regulator of pluripotency and self-renewal in ES cells, has also been shown to accelerate tumorigenesis by promoting the self-renewal and long-term proliferation of lung CSCs 37. Schaal *et al.* found that nicotine induces the expression of the embryonic stem cell factor, Sox2, to regulate CSC properties in NSCLC cells 38. Therefore, CD133, ALDH1A1, Oct4, Nanog, and Sox2 were used as lung CSC markers in this study.

Accumulating evidence has highlighted that the induction of stemness under TS stress is an early step in cancer reprogramming 39, 40. Chronic TS exposure has been shown to induce stem cell features in pancreatic cancer cells *via* PAF1 40, whilst long term TS exposure contributes to the malignant transformation of HBE cells and induces the acquisition of stem cell-like properties 39. In line with previous reports, we showed that chronic CSE exposure increased colony formation, induced epithelial-mesenchymal transition-like changes, increased the expression of lung CSC markers (CD133, ALDH1A1, Oct4, and Nanog), and increased *in vivo* tumor incidence, suggesting that TS promotes CSC-related traits in human bronchial epithelial cells.

Genome-wide occupancy studies have illustrated that ΔNp63α, a well-characterized mediator of stem cell function in numerous tissues, is a lung cancer-related gene. ΔNp63α induction has been shown to increase the levels of stemness markers in basal MCF-7 cells 41 whilst the ΔNp63α-mediated activation of bone morphogenetic protein signaling has been shown to govern stem cell activity in normal and malignant mammary epithelial cells 42. Additionally, ΔNp63α can induce TGF-β-mediated lung cancer metastasis *in vivo* 43; however, the role of ΔNp63α in TS-induced lung stemness remains unknown. This study revealed increased ΔNp63α expression levels in the lung cancer tissues of smokers, with ΔNp63α positively correlated with lung CSC markers in these samples. Moreover, ΔNp63α-overexpressing THBE cells formed more and larger tumorspheres and expressed higher levels of lung CSC markers. In contrast, down-regulating ΔNp63α reduced tumorsphere formation and lung CSC marker levels in sphere-forming cells. These data indicate that ΔNp63α regulates the lung CSC-like properties of TS-transformed HBE tumorsphere cells.

Notch signaling critically participates in the regulation of various types of CSCs, including NSCLC 44, 45. Zhang *et al.* found that Notch signaling was required for the self-renewal of NSCLCs 46, whilst a previous study demonstrated that ΔNp63α stimulates Notch pathway signaling in HC11 cells 47. ΔNp63α has also been shown to induce a stem cell phenotype in the MCF-7 breast carcinoma cell line by upregulating the Notch pathway 48. In this study, Notch activation was clearly observed in TS-transformed HBE sphere-forming cells, whilst suppressing Notch inhibited tumorsphere formation ability and reduced lung CSC marker levels in these sphere-forming cells. Moreover, ΔNp63α overexpression increased the expression of NICD and Hes1 and increased the transcriptional activation of Notch. In contrast, inhibiting ΔNp63α reduced Notch signaling activity. These data indicate that ΔNp63α activates Notch signaling to promote the stemness of TS-transformed HBE tumorsphere cells.

TS-induced lung cancer stemness may occur *via* inflammatory pathways. IL-6 is a pleiotropic cytokine that promotes tumorigenicity and metastasis 49, 50. IL-6-expressing lung cancer cells are known to exist in human lung cancer tissues 51 and IL-6 has also been shown to play an important role in protecting NSCLC CD133+ cells from radiation-induced DNA damage and apoptosis 52. IL-6 secretion was shown to be significantly increased when human bronchial epithelial cells were treated with TS 53. Furthermore, Nelson *et al*. illustrated that exogenous IL-6 treatment enhanced the expression of p63 and induced the proliferation of monolayer keratinocyte cells 22. Therefore, we hypothesized that IL-6 is involved in TS exposure-induced ΔNp63α upregulation and stemness in lung CSCs. Our data show that IL-6 levels were higher in the lung cancer tissues of smokers than in those of non-smokers and that ΔNp63α expression was positively associated with IL-6 levels in these tissues. Moreover, IL-6 promoted ΔNp63α and lung CSC-related marker expression in THBE tumorsphere-forming cells.

Targeting CSCs could be an effective approach for cancer treatment. Evidence suggests that SFN can prevent cancer progression after carcinogen exposure and that SFN exhibits more potent inhibitory effects on CSCs than on other tumor cells or normal cells 54-56. As expected, the cytotoxicity of SFN was lower in HBE cells and non-TS exposed HBE cells than in TS-transformed HBE tumorsphere-forming cells. We showed that SFN efficiently abrogated TS exposure-induced lung CSC-like properties by reducing tumorsphere formation and decreasing CSC marker expression. Furthermore, our data suggest that SFN inhibits the TS-triggered activation of ΔNp63α, IL-6 expression, and the Notch pathway in THBE tumorsphere-forming cells. Moreover, we found that the IL-6/ΔNp63α/Notch axis mediated the suppression of long-term TS exposure-induced lung CSC-like properties by SFN. Finally, we found that administrating SFN inhibited the formation of THBE xenograft tumors in NOD/SCID mice and reduced the levels of IL-6, ΔNp63α, NICD, Hes1, and lung CSC markers in THBE xenograft tumor tissues. Collectively, these data indicate that SFN suppresses TS-induced lung CSC properties.

## Conclusions

In summary, this study revealed that the IL-6/ΔNp63α/Notch axis has an important role in the regulation of TS exposure-induced CSC-like properties and the inhibitory effects of SFN on these cells. Our findings provide an insight into the molecular mechanisms of TS-associated lung cancer and SFN intervention.

## Abbreviations

TS: Tobacco smoke
CSCs: Cancer stem cells
EMT: epithelial-mesenchymal transition
CSE: cigarette smoke extract
IL-6: Interleukin-6
TP63: protein 63
NSCLC: non-small cell lung cancer
HBE cells: human bronchial epithelial cells
NICD: Notch intracellular domain
Hes 1: Hes family BHLH transcription factor 1
SFN: Sulforaphane
THBE: HBE cells exposed to CSE for 55 passages
CHBE: control HBE cells not exposed to CSE

## References

[1] Tonini G, D'Onofrio L, Dell'Aquila E, Pezzuto A (2013). New molecular insights in tobacco-induced lung cancer. Future Oncol.

[2] Schaal CM, Bora-Singhal N, Kumar DM, Chellappan SP (2018). Regulation of Sox2 and stemness by nicotine and electronic-cigarettes in non-small cell lung cancer. Mol Cancer.

[3] Wang J, Chen J, Jiang Y, Shi Y, Zhu J, Xie C (2018). Wnt/beta-catenin modulates chronic tobacco smoke exposure-induced acquisition of pulmonary cancer stem cell properties and diallyl trisulfide intervention. Toxicol Lett.

[4] Hirata N, Yamada S, Sekino Y, Kanda Y (2017). Tobacco nitrosamine NNK increases ALDH-positive cells *via* ROS-Wnt signaling pathway in A549 human lung cancer cells. J Toxicol Sci.

[5] Visvader JE, Lindeman GJ (2008). Cancer stem cells in solid tumours: accumulating evidence and unresolved questions. Nat Rev Cancer.

[6] Rivera C, Rivera S, Loriot Y, Vozenin MC, Deutsch E (2011). Lung cancer stem cell: new insights on experimental models and preclinical data. J Oncol.

[7] Pignon JC, Grisanzio C, Geng Y, Song J, Shivdasani RA, Signoretti S (2013). p63-expressing cells are the stem cells of developing prostate, bladder, and colorectal epithelia. Proc Natl Acad Sci U S A.

[8] Senoo M, Pinto F, Crum CP, McKeon F (2007). p63 is essential for the proliferative potential of stem cells in stratified epithelia. Cell.

[9] Chakrabarti R, Wei Y, Hwang J, Hang X, Andres Blanco M, Choudhury A (2014). ΔNp63 promotes stem cell activity in mammary gland development and basal-like breast cancer by enhancing Fzd7 expression and Wnt signalling. Nat Cell Biol.

[10] Keyes WM, Pecoraro M, Aranda V, Vernersson-Lindahl E, Li W, Vogel H (2011). ΔNp63α is an oncogene that targets chromatin remodeler Lsh to drive skin stem cell proliferation and tumorigenesis. Cell Stem Cell.

[11] Bishop JA, Teruya-Feldstein J, Westra WH, Pelosi G, Travis WD, Rekhtman N (2012). p40 (ΔNp63) is superior to p63 for the diagnosis of pulmonary squamous cell carcinoma. Mod Pathol.

[12] Massion PP, Taflan PM, Jamshedur RS, Yildiz P, Shyr Y, Edgerton ME (2003). Significance of p63 amplification and overexpression in lung cancer development and prognosis. Cancer Res.

[13] Ratovitski EA (2010). LKB1/PEA3/ΔNp63 pathway regulates PTGS-2 (COX-2) transcription in lung cancer cells upon cigarette smoke exposure. Oxid Med Cell Longev.

[14] Licciulli S, Avila JL, Hanlon L, Troutman S, Cesaroni M, Kota S (2013). Notch1 is required for Kras-induced lung adenocarcinoma and controls tumor cell survival *via* p53. Cancer Res.

[15] Li W, Zhou J, Chen Y, Zhang G, Jiang P, Hong L (2017). Cigarette smoke enhances initiation and progression of lung cancer by mutating Notch1/2 and dysregulating downstream signaling molecules. Oncotarget.

[16] Garcia-Arcos I, Geraghty P, Baumlin N, Campos M, Dabo AJ, Jundi B (2016). Chronic electronic cigarette exposure in mice induces features of COPD in a nicotine-dependent manner. Thorax.

[17] Chen PH, Chang H, Chang JT, Lin P (2012). Aryl hydrocarbon receptor in association with RelA modulates IL-6 expression in non-smoking lung cancer. Oncogene.

[18] Ibrahim SA, Hassan H, Vilardo L, Kumar SK, Kumar AV, Kelsch R (2013). Syndecan-1 (CD138) modulates triple-negative breast cancer stem cell properties *via* regulation of LRP-6 and IL-6-mediated STAT3 signaling. Plos One.

[19] Kim SY, Kang JW, Song X, Kim BK, Yoo YD, Kwon YT (2013). Role of the IL-6-JAK1-STAT3-Oct-4 pathway in the conversion of non-stem cancer cells into cancer stem-like cells. Cell Signal.

[20] Sansone P, Storci G, Tavolari S, Guarnieri T, Giovannini C, Taffurelli M (2007). IL-6 triggers malignant features in mammospheres from human ductal breast carcinoma and normal mammary gland. J Clin Invest.

[21] Liu CC, Lin JH, Hsu TW, Su K, Li AF, Hsu HS (2015). IL-6 enriched lung cancer stem-like cell population by inhibition of cell cycle regulators *via* DNMT1 upregulation. Int J Cancer.

[22] Nelson AM, Katseff AS, Ratliff TS, Garza LA (2016). Interleukin 6 and STAT3 regulate p63 isoform expression in keratinocytes during regeneration. Exp Dermatol.

[23] Ge M, Zhang L, Cao L, Xie C, Li X, Li Y (2019). Sulforaphane inhibits gastric cancer stem cells *via* suppressing sonic hedgehog pathway.

[24] Zhu J, Wang S, Chen Y, Li X, Jiang Y, Yang X (2017). miR-19 targeting of GSK3β mediates sulforaphane suppression of lung cancer stem cells. J Nutr Biochem.

[25] Huang YT, Lin X, Liu Y, Chirieac LR, McGovern R, Wain J (2011). Cigarette smoking increases copy number alterations in nonsmall-cell lung cancer. Proc Natl Acad Sci U S A.

[26] Reddel RR, Ke Y, Gerwin BI, McMenamin MG, Lechner JF, Su RT (1988). Transformation of human bronchial epithelial cells by infection with SV40 or adenovirus-12 SV40 hybrid virus, or transfection *via* strontium phosphate coprecipitation with a plasmid containing SV40 early region genes. Cancer Res.

[27] Schiller J, Sabatini L, Bittner G, Pinkerman C, Mayotte J, Levitt M (1994). Phenotypic, molecular and genetic-characterization of transformed human bronchial epithelial-cell strains. Int J Oncol.

[28] Reddel RR, De Silva R, Duncan EL, Rogan EM, Whitaker NJ, Zahra DG (1995). SV40-induced immortalization and ras-transformation of human bronchial epithelial cells. Int J Cancer.

[29] Xu H, Ling M, Xue J, Dai X, Sun Q, Chen C (2018). Exosomal microRNA-21 derived from bronchial epithelial cells is involved in aberrant epithelium-fibroblast cross-talk in COPD induced by cigarette smoking. Theranostics.

[30] Liu Y, Luo F, Wang B, Li H, Xu Y, Liu X (2016). STAT3-regulated exosomal miR-21 promotes angiogenesis and is involved in neoplastic processes of transformed human bronchial epithelial cells. Cancer Lett.

[31] Jin Q, Menter DG, Mao L, Hong WK, Lee HY (2008). Survivin expression in normal human bronchial epithelial cells: an early and critical step in tumorigenesis induced by tobacco exposure. Carcinogenesis.

[32] Tran MN, Choi W, Wszolek MF, Navai N, Lee IL, Nitti G (2013). The p63 protein isoform ΔNp63α inhibits epithelial-mesenchymal transition in human bladder cancer cells: role of MIR-205. J Biol Chem.

[33] Su YJ, Lin WH, Chang YW, Wei KC, Liang CL, Chen SC (2015). Polarized cell migration induces cancer type-specific CD133/integrin/Src/Akt/GSK3β/β-catenin signaling required for maintenance of cancer stem cell properties. Oncotarget.

[34] Jiang F, Qiu Q, Khanna A, Todd NW, Deepak J, Xing L (2009). Aldehyde dehydrogenase 1 is a tumor stem cell-associated marker in lung cancer. Mol Cancer Res.

[35] Nichols J, Zevnik B, Anastassiadis K, Niwa H, Klewe-Nebenius D, Chambers I (1998). Formation of pluripotent stem cells in the mammalian embryo depends on the POU transcription factor Oct4. Cell.

[36] Chen YC, Hsu HS, Chen YW, Tsai TH, How CK, Wang CY (2008). Oct-4 expression maintained cancer stem-like properties in lung cancer-derived CD133-positive cells. Plos One.

[37] Chiou SH, Wang ML, Chou YT, Chen CJ, Hong CF, Hsieh WJ (2010). Coexpression of Oct4 and Nanog enhances malignancy in lung adenocarcinoma by inducing cancer stem cell-like properties and epithelial-mesenchymal transdifferentiation. Cancer Res.

[38] Schaal CM, Bora-Singhal N, Kumar DM, Chellappan SP (2018). Regulation of Sox2 and stemness by nicotine and electronic-cigarettes in non-small cell lung cancer. Mol Cancer.

[39] Liu Y, Luo F, Xu Y, Wang B, Zhao Y, Xu W (2015). Epithelial-mesenchymal transition and cancer stem cells, mediated by a long non-coding RNA, HOTAIR, are involved in cell malignant transformation induced by cigarette smoke extract. Toxicol Appl Pharmacol.

[40] Nimmakayala RK, Seshacharyulu P, Lakshmanan I, Rachagani S, Chugh S, Karmakar S (2018). Cigarette Smoke Induces Stem Cell Features of Pancreatic Cancer Cells *via* PAF1. Gastroenterology.

[41] Amin R, Morita-Fujimura Y, Tawarayama H, Semba K, Chiba N, Fukumoto M (2016). ΔNp63α induces quiescence and downregulates the BRCA1 pathway in estrogen receptor-positive luminal breast cancer cell line MCF7 but not in other breast cancer cell lines. Mol Oncol.

[42] Balboni AL, Hutchinson JA, DeCastro AJ, Cherukuri P, Liby K, Sporn MB (2013). ΔNp63α-mediated activation of bone morphogenetic protein signaling governs stem cell activity and plasticity in normal and malignant mammary epithelial cells. Cancer Res.

[43] Latina A, Viticchie G, Lena AM, Piro MC, Annicchiarico-Petruzzelli M, Melino G (2016). ΔNp63 targets cytoglobin to inhibit oxidative stress-induced apoptosis in keratinocytes and lung cancer. Oncogene.

[44] Zheng Q, Qin H, Zhang H, Li J, Hou L, Wang H (2007). Notch signaling inhibits growth of the human lung adenocarcinoma cell line A549. Oncol Rep.

[45] Chen Y, De Marco MA, Graziani I, Gazdar AF, Strack PR, Miele L (2007). Oxygen concentration determines the biological effects of NOTCH-1 signaling in adenocarcinoma of the lung. Cancer Res.

[46] Zhang Y, Xu W, Guo H, Zhang Y, He Y, Lee SH (2017). NOTCH1 Signaling Regulates Self-Renewal and Platinum Chemoresistance of Cancer Stem-like Cells in Human Non-Small Cell Lung Cancer. Cancer Res.

[47] Kent S, Hutchinson J, Balboni A, Decastro A, Cherukuri P, Direnzo J (2011). DeltaNp63alpha promotes cellular quiescence *via* induction and activation of Notch3. Cell Cycle.

[48] Du Z, Li J, Wang L, Bian C, Wang Q, Liao L (2010). Overexpression of ΔNp63α induces a stem cell phenotype in MCF7 breast carcinoma cell line through the Notch pathway. Cancer Sci.

[49] Lederle W, Depner S, Schnur S, Obermueller E, Catone N, Just A (2011). IL-6 promotes malignant growth of skin SCCs by regulating a network of autocrine and paracrine cytokines. Int J Cancer.

[50] Rojas A, Liu G, Coleman I, Nelson PS, Zhang M, Dash R (2011). IL-6 promotes prostate tumorigenesis and progression through autocrine cross-activation of IGF-IR. Oncogene.

[51] Ogawa H, Koyanagi-Aoi M, Otani K, Zen Y, Maniwa Y, Aoi T (2017). Interleukin-6 blockade attenuates lung cancer tissue construction integrated by cancer stem cells. Sci Rep.

[52] Chen Y, Zhang F, Tsai Y, Yang X, Yang L, Duan S (2015). IL-6 signaling promotes DNA repair and prevents apoptosis in CD133+ stem-like cells of lung cancer after radiation. Radiat Oncol.

[53] Koo JB, Han JS (2016). Cigarette smoke extract-induced interleukin-6 expression is regulated by phospholipase D1 in human bronchial epithelial cells. J Toxicol Sci.

[54] Fan P, Zhang Y, Liu L, Zhao Z, Yin Y, Xiao X (2016). Continuous exposure of pancreatic cancer cells to dietary bioactive agents does not induce drug resistance unlike chemotherapy. Cell Death Dis.

[55] Rausch V, Liu L, Kallifatidis G, Baumann B, Mattern J, Gladkich J (2010). Synergistic activity of sorafenib and sulforaphane abolishes pancreatic cancer stem cell characteristics. Cancer Res.

[56] Kallifatidis G, Labsch S, Rausch V, Mattern J, Gladkich J, Moldenhauer G (2011). Sulforaphane increases drug-mediated cytotoxicity toward cancer stem-like cells of pancreas and prostate. Mol Ther.

